# NEPTUNE. On the seven seas of resilience

**DOI:** 10.1080/10872981.2023.2246782

**Published:** 2023-08-20

**Authors:** Tobias Benedikt Wirthmiller, Beate Ursula Neu, Felix Michael Schmitz, Benny Wohlfarth

**Affiliations:** a Department of Angiology, Bern University Hospital, University of Bern, Bern, Switzerland; b Institute of Medical Education (IML), University of Bern, Bern, Switzerland

**Keywords:** Medical students, resilience, well-being, mental health, 7Cs of resilience

Dear Editor,

It is widely recognized that medical schools face challenges regarding their students’ mental health states [1,2]. Most of our future doctors report a mentally healthy state at the beginning of training and show an increase in emotional imbalances, including anxiety and depression, during their time in medical school [3–5]. With recent events such as the COVID-19 pandemic and concurrent economic challenges [6,7], additional risks of mental health impairment have arisen in medical students around the world [8]. Possible sequelae like an altered or less compassionate attitude towards patients, an increased dropout rate from medical school and ultimately a higher likelihood of attempting suicide [9–11], hold a substantial burden for society, implying a strong need to fortify the resilience of our future workforce to its utmost potential.

While there are a myriad of definitions for psychological resilience, most include the individual’s ability to recover and bounce back from adverse life events [12]. There is an ongoing debate on whether resilience constitutes a stable trait of personality or a dynamic process that varies, depending on the given context [12,13]. Here, we rely on the conception that resilience is – at least partially – a dynamic process that can be influenced by promoting protective factors.

In the following, we propose an adaptation of the 7Cs of resilience (i.e., competence, confidence, connection, character, contribution, coping, and control) from Ginsburg and Jablow [14] as a promising tool for medical schools to promote the mental health of their students. The 7Cs have already been discussed in association with good mental health practices in physicians [15]. However, we remodeled the 7Cs to fit a ‘post’ COVID-19 medical school scenario and give practical advice to students and faculty alike with our NEPTUNE model.

## N–nurturing certainty (confidence)

COVID-19 posed a challenge to university curricula, leaving many medical students uncertain about the demands of residency [16]. However, virtual courses have emerged as effective tools for developing certain practical skills, such as handling calls, to increase student confidence [17]. Given faculties’ limitations in space and personnel, we suggest leveraging the scalability of virtual courses to free on-site resources for training aspects that cannot be practiced virtually.

## E–engaging expertise (competence)

Although simulations can be beneficial to develop confidence [18], for certain scenarios, hands-on experience is more likely to increase student readiness for clinical situations [19] and reduces the stress of transitioning to a junior doctor position [20]. To improve clinical competence while ensuring patient safety, we suggest fostering longitudinal training models, as continuity reinforces the bond and commitment between students and supervisors, resulting in greater trust for competence in independent patient care [21] and ultimately expertise.

## P–pursuing relationships (connection)

Social support and perceived group cohesion are powerful factors to buffer the effects of stressful events on mental health [22] while also having a benefit on learning [23]. Consequently, during the pandemic, medical students experienced less stress, when they felt stronger social support [24,25]. We suggest fostering more stable learning cohorts during studies to promote comradery and friendships among students.

## T–training integrity (character)

During medical training, many students witness unethical behavior of other healthcare personnel, often leaving them with a feeling of guilt or complicity [26]. These experiences are related to poorer mental health and promote more cynical attitudes in students, which could affect the quality of patient treatment [11]. We propose the consequent sensibilization of personnel involved in medical training and the implementation of programs, where students can discuss moral issues in medicine, share their experiences, and discuss potential resorts.

## U–understanding impact (contribution)

During the pandemic, medical students volunteered to support the healthcare system struggling with high demands on medical services [27]. Many participants reported benefits of taking over responsibility and contributing to a team [28]. Faculties should therefore support student-driven community outreach projects, e.g., by 1. providing access to expert content consultation, 2. offering courses on how to organize voluntary work and 3. contributing resources for meetings and events.

## N–navigating challenges (coping)

In stressful situations, it is important to fulfill tasks while ensuring mental integrity. Strategies focused on problem-solving or -acceptance proved to have positive implications on students’ mental health, while drug-abuse and problem-avoidance had a negative impact [29]. Faculties should support their students by offering problem-oriented strategies, e.g., through courses on effective exam preparation, enhancing the students’ ability to devise realistic timetables for these periods. Furthermore, meditation programs like the mindfulness-based stress reduction [30] can foster the well-being of medical students [31] and should be offered by faculty more often.

## E–encouraging autonomy (control)

While autonomy in medical decision-making is asked for in physicians [32], medical students are often faced with a highly structured learning environment, limited by external factors and regulations that hinder the possibility of ‘creating and shaping’ [33]. Faculties could alleviate the feeling of narrow control by supporting learners with the flexibility to shape their own careers and learning paths.
